# Detection and drug resistance profile of *Escherichia coli* from subclinical mastitis cows and water supply in dairy farms in Saraburi Province, Thailand

**DOI:** 10.7717/peerj.3431

**Published:** 2017-06-13

**Authors:** Woranich Hinthong, Natapol Pumipuntu, Sirijan Santajit, Suphang Kulpeanprasit, Shutipen Buranasinsup, Nitat Sookrung, Wanpen Chaicumpa, Pisinee Aiumurai, Nitaya Indrawattana

**Affiliations:** 1 Faculty of Medicine and Allied Health, HRH Princess Chulabhorn College of Medical Science, Bangkok, Thailand; 2 Department of Microbiology and Immunology, Faculty of Tropical Medicine, Mahidol University, Bangkok, Thailand; 3 Faculty of Veterinary Sciences, Mahasarakham University, Mahasarakham, Thailand; 4 Department of Pre-clinic and Applied Animal Science, Faculty of Veterinary Medicine, Mahidol University, Nakornpathom, Thailand; 5 Department of Research and Development, Faculty of Medicine Siriraj Hospital, Mahidol University, Bangkok, Thailand; 6 Center of Excellence on Therapeutic Proteins and Antibody Engineering, Department of Parasitology, Faculty of Medicine Siriraj Hospital, Mahidol University, Bangkok, Thailand

**Keywords:** Subclinical bovine mastitis, *Escherichia coli*, Antibiotic resistance, Extend spectrum beta-lactamase

## Abstract

Subclinical mastitis is a persistent problem in dairy farms worldwide. Environmental *Escherichia coli* is the bacterium predominantly responsible for this condition. In Thailand, subclinical mastitis in dairy cows is usually treated with various antibiotics, which could lead to antibiotic resistance in bacteria. *E. coli* is also a reservoir of many antibiotic resistance genes, which can be conveyed to other bacteria. In this study, the presence of *E. coli* in milk and water samples was reported, among which enteropathogenic *E. coli* was predominant, followed by enteroaggregative *E. coli* and enterohemorrhagic *E. coli*, which was found only in milk samples. Twenty-one patterns of antibiotic resistance were identified in this study. Ampicillin- and carbenicillin-resistant *E. coli* was the most common among the bacterial isolates from water samples. Meanwhile, resistance to ampicillin, carbenicillin, and sulfamethoxazole-trimethoprim was the pattern found most commonly in the* E. coli* from milk samples. Notably, only the *E. coli* from water samples possessed ESBL phenotype and carried antibiotic resistance genes, *bla*_TEM_ and *bla*_CMY-2_. This indicates that pathogenic* E. coli* in dairy farms is also exposed to antibiotics and could potentially transfer these genes to other pathogenic bacteria under certain conditions.

## Introduction

In dairy farms, mastitis is a persistent problem resulting in economic losses and premature culling of cows. *Staphylococcus aureus* (*S. aureus*) is considered a major causative pathogen which is a threat to farmers, although easily identifiable, whereas other gram negative bacteria is overlooked or not considered to be a cause for concern by farmers. Subclinical mastitis, which is defined as a somatic cell count (SCC) of >200,000 cells/mL in milk, is usually caused by gram negative bacteria, such as *Escherichia coli* (*E. coli*), *Klebsiella pneumoniae*, and *Serratia marcescen*s (Schukken et al., 2012; Azevedo et al., 2016). These bacteria are commonly found in environmental settings, such as bedding, clothes, farmers’ hands, and water used on farms (Perkins et al., 2009; Iraguha, Hamudikuwanda & Mushonga, 2015; Azevedo et al., 2016). Among gram negative bacteria, *E. coli* is the most notable cause of mastitis. *E. coli* was found to usually infected mammary gland of cows parturition and early lactation period which could lead to local and acute mastitis (Burvenich et al., 2003). In a study in Portugal, *E. coli* was found to be the second most common bacteria after non-coagulative staphylococci found in bulk tank milk (Azevedo et al., 2016). In Uruguay, *E. coli* was second only to *S. aureus* in bovine subclinical mastitis cases (Gianneechini et al., 2002), whereas in China, it was one of the leading types of coliform bacteria found in milk from cows with subclinical mastitis (Memon et al., 2013; Wang et al., 2015).

The treatment of bovine subclinical mastitis usually depends on the severity of the symptoms. In Thailand, the disease is usually treated with antibiotics or the infected cows are culled. Antibiotics are also used for prevention in some farms. However, this can lead to bacteria developing resistance to them. For example, increased resistance to antibiotics in *S. aureus* in the form of oxacillin- or gentamicin-resistant strains was reported in Thailand due to their excessive use (Suriyasathaporn, 2011; Suriyasathaporn et al., 2012). Despite this background, there is little information on antibiotic resistance and drug resistance genes in other bacteria related to bovine mastitis in Thailand. *E. coli* can be antibiotic-resistant as it is also exposed to antibiotics from wastewater from farms. Furthermore, *E. coli* that carries resistance genes can transfer those genes to other pathogenic bacteria (Hu et al., 2016). The discovery of antibiotic resistance in *E. coli* isolates from farms could possibly show the trend or specific characteristic of antibiotic resistance and facilitate better prevention or the more effective treatment for mastitis on dairy farm. This study was thus conducted to detect *E. coli* from water sources and milk from cows with subclinical mastitis, and their antibiotic resistance patterns.

## Materials and Methods

### Sample collection

All procedures performed in this study are in accordance with the ethical standards of the Faculty of Tropical Medicine–Animal Care and Use Committee (FTM-ACUC), Mahidol University, Thailand (protocol no. 002-2016). Water and milk samples were collected from 17 dairy farms in Saraburi Province, Thailand, where agriculture and livestock are the main source of income of the people. A total of 35 water samples were collected in 500-ml sterile bottles from drinking water for cows in a milking area and also from washing water. Thirty-eight milk samples were collected in sterile falcon tubes from cows with subclinical mastitis, which had an SCC of >200,000 cells/ml in milk, after the teats had been disinfected with 70% ethanol and 4–5 streams of milk had been removed. Both water and milk samples were stored at 4 °C and transported to the laboratory within 24 h for the experiment.

### Bacterial isolation

Each water and milk sample was centrifuged at 6,000 rpm for 10 min, and the precipitant was subjected to 10-fold dilution and spread on MacConkey agar (Becton, Dickinson, and Company). Suspected *E. coli* lactose-fermenting colonies (pink colonies) were subjected to gram staining and standard biochemical tests, including triple sugar iron agar, lysine decarboxylase, ornithine decarboxylase/deaminase, motility, and indole production tests.

### Antibiotic susceptibility tests

All *E. coli* isolates were subjected to antibiotic susceptibility tests following the Clinical and Laboratory Standards Institute (CLSI) guidelines (Clinical and Laboratory Standards Institute, 2016). The antimicrobial disks used in the experiment included 10 µg ampicillin (≤13 mm), 100 µg piperacillin (≤17 mm), 10 µg carbenicillin (≤17 mm), 20 µg amoxicillin-clavulanic acid (≤13 mm), 30 µg cefepime (≤14 mm), 30 µg cefotaxime (≤22 mm), 30 µg ceftriaxone (≤19 mm), 30 µg ceftazidime (≤17 mm), 75 µg cefoperazone (≤15 mm), 30 µg cefuroxime (≤14 mm), 10 µg imipenem (≤19 mm), 10 µg meropenem (≤19 mm), 10 µg gentamicin (≤12 mm), 30 µg amikacin (≤14 mm), 15 µg tigecycline (≤14 mm), 5 µg ciprofloxacin (≤15 mm), 10 µg norfloxacin (≤12 mm), and 23.75 µg trimethoprim–sulfamethoxazole (≤10 mm) (Oxoid). *E. coli* strain ATCC 25922 was used as a control in this experiment.

Extended spectrum β-lactamase (ESBL) production was tested by double disk synergy (DDS) method modified from Clinical and Laboratory Standards Institute (2012). The test uses 30 µg antibiotic disks of cefepime, cefotaxime ceftriaxone, ceftazidime, and cefuroxime. The antibiotic disks were placed on the *E. coli* spreaded MHA culture plate, 30 mm (center to center) from the amoxicillin-clavulanic acid (30 µg) disk. Plates were incubated at 37 °C overnight and observed for the presence of an extended spectrum beta-lactamase (ESBL) phenotype by an extension of the edge of inhibition zone of antibiotic disks toward the amoxicillin-clavulanic acid.

### Gene detection by polymerase chain reaction

All *E. coli* isolates from both water and milk samples were determined using specific gene and plasmid, and the isolates that showed resistance to antibiotics were selected and subjected to PCR to investigate their drug resistance genes. The bacteria were cultured in 1.5 ml of tryptic soy broth (Oxoid) and incubated overnight; they were then harvested and centrifuged for 10 min at 6,000 rpm. The pellet was resuspended with 800 µl of sterile distilled water, boiled for 10 min, centrifuged at 6,000 rpm for 10 min, and then the supernatant was collected for use as a DNA template in PCR. PCR primers, conditions, and positive control strains for the detection of target gene and drug resistance genes are presented in Tables 1 and 2. All PCR reactions with a total volume of 25 µl were performed in 1× Taq buffer, 1 mM MgCl_2_, 0.2 mM dNTP, 1 µM of each of the forward and reverse primers, and 2 units of *Taq* DNA polymerase (Thermo Scientific). The PCR amplicon was subjected to 1.5% agarose gel electrophoresis in TAE buffer. For gene amplification with no reference control, the PCR product from positive samples was subjected to nucleotide sequencing and sequence analysis for gene confirmation.

**Table 1:** Primers and PCR conditions used for virulence gene detection.

Target genes	Positive control	Sequences (5′–3′)	Annealing temperature (°C)	Product size (bp)	References
Heat-labile toxin (*lt*)	ETEC	tctctatgcatacggag ccatactgattgccgcaatt	55	322	Deng et al. (1996)
Hest-stable toxin (*st*)	ETEC	tgctaaaccagtagagtcttcaaaa gcaggcttacaacacaattcacagcag	55	138	Mercado et al. (2011)
Shiga-like enterotoxins 1 (*evt*)	EHEC	caacactggatgatctcag ccccctcaactgctaata	55	349	Khan et al. (2002)
Shiga-like enterotoxins 2 (*evs*)	EHEC	atcagtcgtcactcactggt ctgctgtcacagtgacaaa	55	110	Khan et al. (2002)
Transcriptional activator of the aggregative adherence fimbriae (*agg*R)	EAEC 17-2	ctaattgtacaatcgatgta atgaagtaattcttgaat	55	308	Nataro et al. (1994)
*pCVD*432 plasmid	EAEC 17-2	ctggcgaaagactgtatcat caatgtatagaaatccgctgtt	55	630	Aranda, Fagundes-Neto & Scaletsky (2004)
Intimin (*eae*A)	Plasmid-*eae*A	aaacaggtgaaactgttgcc tctcgcctgatagtgtttggta	55	350	Yu & Kaper (1992)
Bundle-forming pilus (*bfp*A)	–	aatggtgcttgcgcttgctgc gccgctttatccaacctggta	57	326	Zhang et al. (2016)

**Notes.**

ETEC enterotoxigenic *E. coli* EHEC enterohemorrhagic *E. coli* EAEC enteroaggregative *E. coli*

**Table 2:** Primers and PCR conditions used for antibiotic resistance gene detection.

Drug resistance genes	Positive control	Sequences (5′–3′)	Annealing temperature (°C)	Product size (bp)	References
***Beta-lactams***					
*bla*_TEM_	–	ttaactggcgaactacttac gtctatttcgttcatccata	60	247	Kozak et al. (2009)
*bla*_SHV_	–	aggattgactgccttttg atttgctgatttcgctcg	60	393	Kozak et al. (2009)
*bla*_CMY-2_	–	gacagcctctttctccaca tggacacgaaggctacgta	60	1,000	Kozak et al. (2009)
***Aminoglycosides***					
*aac*(3)-IIa	–	cggaaggcaataacggag tcgaacaggtagcactgag	60	740	Soleimani et al. (2014)
*aac*(3)-IV	–	gtgtgctgctggtccacagc agttgacccagggctgtcgc	60	627	Maynard et al. (2004)
*aad*A	–	cccctggagagagcgagatt cgtggctggctcgaagatac	60	152	Our study
*aad*B	–	gaggagttggactatggatt cttcatcggcatagtaaaag	60	208	Kozak et al. (2009)
***Quinolone***					
*qnr*A	–	agaggatttctcacgccagg tgccaggcacagatcttgac	60	580	Cattoir et al. (2007)
*qnr*B	–	ggcattgaaattcgccactg tttgctgctcgccagtcgaa	60	264	Cattoir et al. (2007)
*qnr*S	–	gcaagttcattgaacagggt tctaaaccgtcgagttcggcg	60	428	Cattoir et al. (2007)

### Serotyping

*E. coli* isolates with virulence genes were serotyped using Serosystem (Serosystem, Clinag, Thailand) to identify O and H antigens present on the surface of the pathogenic *E. coli* isolates with slide agglutination test. The experiment was performed following the manufacturer’s protocol. EAEC, EHEC, EPEC, and ETEC strains were used as positive control in the experiment.

## Results

### *E. coli* isolation and antibiotic resistance patterns

**Figure 1:**
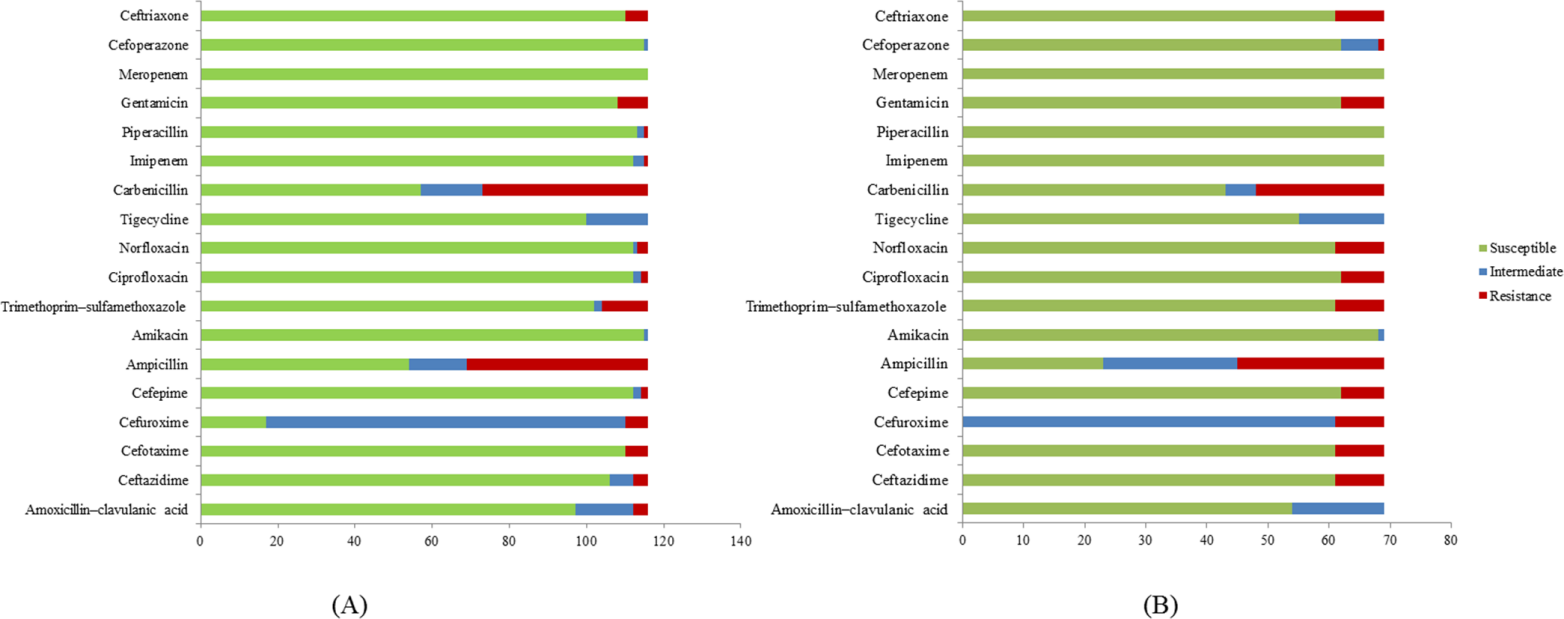
Antibiotic susceptibility test results. (A) *E. coli* isolates from water samples. (B) *E. coli* isolates from milk samples.

A total of 185 *E. coli* isolates were collected from water (116 isolates) and milk (69 isolates) samples and subjected to antibiotic susceptibility tests. Among these isolates, a total of 77 (51 isolates from water and 26 isolates from milk samples) showed resistance to at least one of the antibiotics use in the experiment. Penicillin-resistant *E. coli* (71/77, 92.2%) was found to be the largest group in this study followed by folate pathway inhibitor-resistant *E. coli* (20/77, 26%). *E. coli* resistant to cephems (14/77, 18.2%), aminoglycosides (14/77, 18.2%), *β*-lactamase inhibitor combination (4/77, 5.2%), fluoroquinolone (12/77, 14.3%), and carbapenem (1/77, 1.3%) were also found. Among antibiotic resistant *E. coli*, 84.31% (43/51) of *E. coli* found in water samples are multidrug resistance and 84.61% (22/26) in milk samples (Table 3). The antibiotic patterns could be divided into 21 types, as shown in Table 3. We also found the ESBL phenotype (12/185, 6.5%) in six *E. coli* isolates each from water and milk samples. The antibiotics that the *E. coli* strains are susceptible to are as shown in Fig. 1.

**Table 3:** Distribution of antibiotic resistance phenotypic patterns of *E. coli* isolates.

Resistance pattern	Phenotypic resistance	Number of resistant *E. coli* isolates	
		Water samples (*n* = 51)	Milk samples (*n* = 26)
I	AMC	1 (1.9%)	0 (0%)
II	AMC, AMP, CAR	2 (3.9%)	0 (0%)
III	AMC, AMP, CAR, IPM	1 (1.9%)	0 (0%)
IV	AMP	3 (5.8%)	2 (7.6%)
V	AMP, CAR	26 (50.9%)	0 (0%)
VI	AMP, CAR, CAZ, CN, CRO, CTX, CXM	2 (3.9%)	0 (0%)
VII	AMP, CAR, CAZ, CN, CRO, CTX, CXM, FEP	0 (0%)	6 (23.0%)
VIII	AMP, CAR, CAZ, CN, CRO, CTX, CXM, FEP, SCF	0 (0%)	1 (3.8%)
IX	AMP, CAR, CAZ, CN, CRO, CTX, CXM, FEP, SXT	1 (1.9%)	0 (0%)
X	AMP, CAR, CAZ, CN, CRO, CTX, CXM, SXT	1 (1.9%)	0 (0%)
XI	AMP, CAR, CIP, CN, CRO, CTX, CXM, FEP, SXT	1 (1.9%)	0 (0%)
XII	AMP, CAR, CIP, NOR	0 (0%)	7 (26.9%)
XIII	AMP, CAR, CIP, NOR, SXT	1 (1.9%)	0 (0%)
XIV	AMP, CAR, CN, CRO, CTX, CXM, SXT	1 (1.9%)	0 (0%)
XV	AMP, CAR, NOR	1 (1.9%)	0 (0%)
XVI	AMP, CAR, SXT	6 (11.7%)	7 (26.9%)
XVII	AMP, CAZ, CRO, CTX, CXM	0 (0%)	1 (3.8%)
XVIII	CN	1 (1.9%)	0 (0%)
XIX	NOR	1 (1.9%)	1 (3.8%)
XX	SXT	1 (1.9%)	1 (3.8%)
XXI	TZP	1 (1.9%)	0 (0%)

**Notes.**

AMP ampicillin TZP piperacillin CAR carbenicillin AMC amoxicillin–clavulanic acid FEP cefepime CTX cefotaxime CRO ceftriaxone CAZ ceftazidime SCF cefoperazone CXM cefuroxime IPM imipenem MEM meropenem CN gentamicin AK amikacin TGC tigecycline CIP ciprofloxacin NOR norfloxacin SXT trimethoprim–sulfamethoxazole

### Specific gene and drug resistance gene detection and serotyping

**Figure 2:**
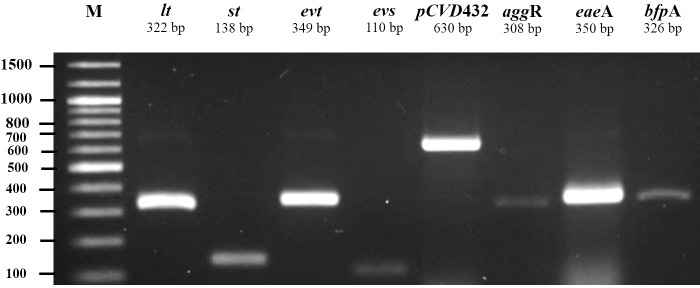
Agarose gel electrophoresis of 1% agarose of the amplification products of virulence genes and plasmid for ETEC (*lt, st*), EHEC (*evt, evs*), EAEC (*pCVD*432, *agg*R), and EPEC (*eae*A, *bfp*A). M, DNA Marker.

**Figure 3:**
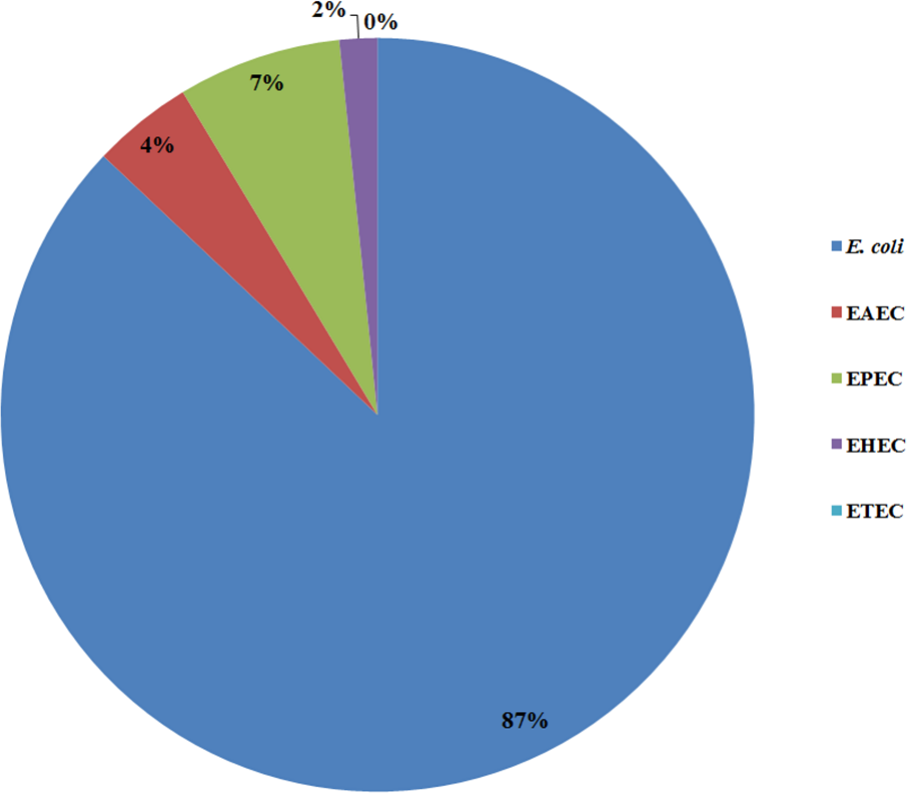
Prevalence of pathogenic *E. coli* detected from water and milk samples.

All 185 *E. coli* isolates from both water and milk samples were also subjected to an analysis of the virulence genes and plasmid for EAEC, EHEC, EPEC, and ETEC (*agg* R and *pCVD*432, *evt* and *evs*, *eae*A and *bfp*A, and *lt* and *st*, Fig. 2). Among the bacterial isolates, 24 (24/185, 12.97%) showed positive results for gene detection by PCR, with *bfp*A positive isolates, EPEC forming the majority (13/185, 7.02%) followed by *pCVD*432 positive isolates, EAEC (8/185, 4.32%) and *evt* positive isolates, EHEC (3/185, 1.62%) (Fig. 3). All EPEC *E. coli* isolates were from water samples. Among them, the bacteria presented different serotypes, namely, O124:K62 (2/13, 15.4%), O111:K58 (2/13, 15.4%), O128:K67 (1/13, 7.7%), O78:K80 (1/13, 7.7%), and O86:K61 (1/13, 7.7%). EAEC isolated from milk samples possessed the O18aO18C:K77 serotype (6/8, 75%) and those from water samples possessed the O112aO112c:K66 serotype (1/8, 12.5%), whereas one isolate could not be serotyped. EHEC isolates from milk samples possessed the O114:K serotype (2/3, 66.7%), whereas positive isolates from water could not be serotyped (Table 4).

The bacterial antibiotic-resistant isolates (77 isolates) were investigated for drug resistance genes (*β*-lactam: *bla*_TEM_, *bla*_SHV_, *bla*_CMY-2_; aminoglycoside: *aac*(3)-IIa, *aac*(3)-IV, *aad*A, *aad*B; quinolone: *qnr*A, *qnr*B, and *qnr*S) using PCR. The results showed that one *pCVD*432 positive *E. coli* and one *bfpA* positive *E. coli* isolates possessed *bla*_CMY-2_ and *bla*_TEM_, respectively. We also found one *pCVD*432 positive isolate with the ESBL phenotype that carried both *bla*_TEM_ and *bla*_CMY-2_. In non-pathogenic *E. coli* isolates, 43 (43/77, 55.9%) isolates were found to possess various antibiotic resistance genes (Table 5). The most common resistant gene found was *bla*_TEM_ (38/62, 61.3%) followed by *bla*_CMY-2_ (16/62, 25.8%) and *aac*(3)IIa (3/62, 4.9%). Other resistance genes carried by non-pathogenic isolates were *aad*A (2/62, 3.3%) and *bla*_SHV_(2/62, 3.3%). None of the *E. coli* isolates carried quinolone resistance genes.

**Table 4:** Target genes, serotyping, antibiotic resistance pattern, resistant gene profile, and ESBL phenotype of pathogenic *E. coli* isolates.

Isolate number	Origin	Target gene	Serotype	Antibiotic resistance pattern	Resistance gene profile	ESBL phenotype
M-W910-1 LF1	Water	*bfp*A	^a^	^*^	−	−
M-W910-1 LF3	Water	*bfp*A	^a^	^*^	^−^	^−^
M-W1010-1 LF1	Water	*bfp*A	^a^	V	*bla*_TEM_	^−^
M-W1110-1 LF3	Water	*bfp*A	^a^	XX	^−^	^−^
M-W1110-1 LF5	Water	*bfp*A	O124:K62	^*^	^−^	^−^
M-W1110-1 LF6	Water	*bfp*A	O111:K58	^*^	^−^	^−^
M-W12UD LFB6	Water	*bfp*A	O111:K58	I	^−^	^−^
M-W13UD LFB6	Water	*bfp*A	O128:K67	^*^	^−^	^−^
M-W13UD LFB7	Water	*bfp*A	O124:K62	^*^	^−^	^−^
M-W13UD LFB10	Water	*bfp*A	O78:K80	^*^	^−^	^−^
M-W15UD LFB4	Water	*bfp*A	^a^	V	^−^	^−^
M-W22UD LF9	Water	*pCVD*432	^a^	VI	*bla*_CMY-2_, *aac*(3)IIa	^−^
M-W23UD LF2	Water	*pCVD*432	O112aO112c:K66	VI	*bla*_TEM_, *bla*_CMY-2_, *aac*(3)IIa*, aad*A	^+^
M-W32UD LF1	Water	*bfp*A	^a^	V	^−^	^−^
M-W33UD LF1	Water	*bfp*A	O86:K61	^*^	^−^	^−^
M-M10UD LFB4	Milk	*evt*	O114:K	^*^	^−^	^−^
M-M10UD LFB5	Milk	*evt*	O114:K	^*^	^−^	^−^
M-M35UD LFB2	Milk	*pCVD*432	O18aO18c:K77	VII	^−^	^+^
M-M35UD LFB3	Milk	*pCVD*432	O18aO18c:K77	VII	^−^	^+^
M-M35UD LFB4	Milk	*pCVD*432	O18aO18c:K77	VII	^−^	^+^
M-M35UD LFB5	Milk	*pCVD*432	O18aO18c:K77	VIII	^−^	^+^
M-M35UD LFB6	Milk	*pCVD*432	O18aO18c:K77	VII	^−^	^+^
M-M35UD LFB7	Milk	*pCVD*432	O18aO18c:K77	VII	^−^	^+^
M-M37UD LFB4	Milk	*evt*	^a^	XVI	^−^	^−^

**Notes.**

a Not typable.

* Susceptible.

+ Positive.

− Negative.

**Table 5:** Target genes, serotyping, antibiotic resistance pattern, resistant gene profile, and ESBL phenotype of non-pathogenic *E. coli* isolates.

Isolate number	Origin	Target gene	Serotype	Antibiotic resistance pattern	Resistance gene profile	ESBL phenotype
M-W610-1 LF1	Water	^−^	^b^	V	*bla*_TEM_	^−^
M-W910-1 LF2	Water	^−^	^b^	IV	*bla*_CMY-2_	^−^
M-W1110-1 LF9	Water	^−^	^b^	XVI	*bla*_TEM_	^−^
M-W12UD LFB6	Water	^−^	^b^	I	*bla*_CMY-2_	^−^
M-W16UD LF1	Water		^b^	V	*bla*_TEM_, *bla*_SHY_, *bla*_CMY-2_	^−^
M-W16UD LF2	Water	^−^	^b^	V	*bla*_TEM_, *bla*_CMY-2_	^−^
M-W1910-1 LF6	Water	^−^	^b^	V	*bla*_TEM_	^−^
M-W20UD LF6	Water	^−^	^b^	V	*bla*_TEM_, *bla*_CMY-2_	^−^
M-W20UD LF9	Water	^−^	^b^	V	*bla*_TEM_, *bla*_CMY-2_	^−^
M-W22UD LF3	Water	^−^	^b^	XIV	*bla*_TEM_, *bla*_CMY-2_	^+^
M-W22UD LF7	Water	^−^	^b^	X	*bla*_TEM_, *bla*_CMY-2_, *aac*(3)IIa	^+^
M-W24UD LF1	Water	^−^	^b^	V	*bla*_TEM_	^−^
M-W24UD LF3	Water	^−^	^b^	XI	*bla*_TEM_, *bla*_CMY-2_, *aac*(3)IIa, *aad*A	^+^
M-W24UD LF4	Water	^−^	^b^	V	*bla*_TEM_	^−^
M-W24UD LF5	Water	^−^	^b^	XVI	*bla*_TEM_	^−^
M-W24UD LF7	Water	^−^	^b^	^*^	*bla*_TEM_, *bla*_CMY-2_	^−^
M-W26UD LF8	Water	^−^	^b^	IV	*bla*_TEM_	^−^
M-W27UD LF4	Water	^−^	^b^	XVI	*bla*_TEM_	^−^
M-W28UD LF1	Water	^−^	^b^	XV	*bla*_TEM_	^−^
M-W28UD LF5	Water	^−^	^b^	V	*bla*_SHV_, *bla*_CMY-2_	^−^
M-W28UD LF7	Water	^−^	^b^	V	*bla*_TEM_, *bla*_CMY-2_	^−^
M-W29UD LF1	Water	^−^	^b^	IX	*bla*_TEM_, *bla*_CMY-2_, *aac*(3)IIa, *aad*A	^+^
M-W29UD LF10	Water	^−^	^b^	XVI	*bla*_TEM_	^−^
M-W31UD LF6	Water	^−^	^b^	V	*bla*_TEM_	^−^
M-W33UD LF2	Water	^−^	^b^	V	*bla*_TEM_	^−^
M-W33UD LF6	Water	^−^	^b^	V	*bla*_TEM_	^−^
M-W33UD LF9	Water	^−^	^b^	V	*bla*_TEM_	^−^
M-W33UD LF10	Water	^−^	^b^	V	*bla*_TEM_	^−^
M-W34UD LF3	Water	^−^	^b^	V	*bla*_TEM_	^−^
M-W34UD LF7	Water	^−^	^b^	XIII	*bla*_TEM_	^−^
M-M1610-1 LFB2	Milk	^−^	^b^	IV	*bla*_TEM_	^−^
M-M37UD LFB1	Milk	^−^	^b^	XVI	*bla*_TEM_	^−^
M-M37UD LFB3	Milk	^−^	^b^	XVI	*bla*_TEM_	^−^
M-M37UD LFB5	Milk	^−^	^b^	XVI	*bla*_CMY-2_	^−^
M-M37UD LFB6	Milk	^−^	^b^	XVI	*bla*_TEM_, *bla*_CMY-2_	^−^
M-M37UD LFB8	Milk	^−^	^b^	XVI	*bla*_CMY-2_	^−^
M-M38UD LFB1	Milk	^−^	^b^	XII	*bla*_TEM_	^−^
M-M38UD LFB2	Milk	^−^	^b^	XII	*bla*_TEM_	^−^
M-M38UD LFB3	Milk	^−^	^b^	XII	*bla*_TEM_	^−^
M-M38UD LFB4	Milk	^−^	^b^	XII	*bla*_TEM_	^−^
M-M38UD LFS2	Milk	^−^	^b^	XII	*bla*_TEM_	^−^
M-M38UD LFS3	Milk	^−^	^b^	XII	*bla*_TEM_	^−^
M-M38UD LFS4	Milk	^−^	^b^	XII	*bla*_TEM_	^−^

**Notes.**

b Not serotype.

* Susceptible.

+ Positive.

− Negative.

## Discussion

*E. coli* is known to be the most common gram negative bacteria that potentially causes subclinical mastitis and exhibits antibiotic resistance. However, pathogenic *E. coli* in the environment has often been overlooked. Many studies have reported the presence of *E. coli* among subclinical mastitis cases in dairy farms in many regions of the world, particularly in developing countries, such as Uruguay, Turkey, Brazil, Ethiopia, Mexico, and China (Gianneechini et al., 2002; Guler & Gunduz, 2007; Fernandes et al., 2011; Haftu et al., 2012; Abera et al., 2012; Olivares-Perez et al., 2015; Wang et al., 2015). This study demonstrated the existence of pathogenic *E. coli* in environmental sources and also in milk from cows with subclinical mastitis by detecting specific genes associated with the pathogenic types of this species. *bfp*A-positive *E. coli* was found to be the most common strain of pathogenic *E. coli* residing in water sources. *pCVD*432-positive isolate was found in both water and milk samples. *evt-*positive *E. coli* was the least common and was only identified in milk samples; it was not present in any of the water samples. In this study, EPEC possessed only *bfp*A, which encodes bundle-forming pili that are a specific characteristic of EPEC (Cleary et al., 2004). The presence of EPEC in water sources in dairy farms could lead to intramammary infection of cows. A study by Dopfer, Nederbragt & Almeida (2001) also reported the isolation of *bfp*A-positive EPEC from persistent cases of bovine mastitis (Dopfer, Nederbragt & Almeida, 2001). Although none of the *E. coli* isolates was positive for *eae*A in this study, there are reports of the presence of *eae*A-positive EPEC among *E. coli* found in cows with mastitis in Brazil and Turkey (Correa & Marin, 2002; Guler & Gunduz, 2007). However, in Iran, *eae*A-positive *E. coli* was not found in clinical mastitis cases (Ghanbarpour & Oswald, 2010). This indicates that *bfp*A- and *eae*A-positive EPEC may be distributed unevenly across the globe. EAEC (*pCVD*432-positive isolates) was found in both water and milk samples. However, the serotypes of those isolates differed. This may indicated different sources of EAEC in water and infected cows. The results also designated that EAEC may be an epidemic strain in dairy farms in Saraburi Province, and EAEC and EPEC could be causative agents of mastitis considering their potential infection through water in farms. EHEC was the least common group found only in milk samples in this study and positive only for *evt* (shiga-toxin 1-encoding gene). These results raise concerns regarding the bacterial distribution to nearby areas via the contaminated water which workers should be aware of. Studies by Lira, Macedo & Marin (2004) and Momtaz (2010) also reported shiga-toxin 1-producing *E. coli* from cases of subclinical mastitis in cows in Brazil and Iran. Momtaz et al. (2012) later reported that shiga-toxin 1-producing *E. coli* was the most common type of *E. coli* in milk samples from cows with subclinical mastitis in Iran (Momtaz et al., 2012). These results also correlated with many studies on clinical cases of bovine mastitis. For example, Momtaz et al. (2012) reported the presence of EHEC with shiga-toxin 1-encoding gene as the most common virulence gene in milk samples from cases with subclinical and clinical mastitis (Momtaz et al., 2012), which also correlated with the study by Suojala et al. (2011), in which shiga-toxin 1-encoding gene was among the most common virulence genes found in clinical cases of bovine mastitis (Suojala et al., 2011).

Among the 21 antibiotic resistance patterns, the most common pattern found in *E. coli* from water sources was pattern V (ampicillin and carbenicillin resistance), followed by pattern XVI (ampicillin, carbenicillin, gentamicin, ceftriaxone, cefotaxime, and trimethoprim/sulfamethoxazole resistance). Among the antibiotic patterns in the *E. coli* from milk, pattern XII (ampicillin, carbenicillin, ciprofloxacin, and norfloxacin resistance) and XVI were the most common. This may indicate that *E. coli* in milk could potentially derive from water or other environmental sources. A study by Sayah et al. (2005) also reported the difference in antibiotic resistance patterns between *E. coli* isolated from farm water and fecal samples (Sayah et al., 2005). Our results call for a more cautious approach with antibiotics usage in dairy farms in the Saraburi province area, since the antibiotics that the *E. coli* isolates were susceptible to are from the high generation cephalosporin and *β*-lactam classes which are normally used for the treatment of drug-resistance bacteria.

In another study, Geser, Stephan & Hachler (2012) reported on ESBL-positive *E. coli* in milk from cows with mastitis Geser, Stephan & Hachler (2012), and ESBL-producing *E. coli* was shown to be able to spread from infected animals to the environment, such as air and slurry, as reported in a pig farm in Germany (Von Salviati et al., 2015). In this study, EAEC was the only pathogenic strain that possessed the ESBL phenotype. Notably, only the ESBL-producing EAEC isolates from water samples contained antibiotic resistance genes (*bla*_TEM_ and *bla*_CMY-2_). The results also correlate with the study by Franz et al. (2015), who reported that EAEC found in surface water and wastewater dominates over other strains of pathogenic *E. coli* in terms of possessing the ESBL phenotype (Franz et al., 2015). In this study, we found that non-pathogenic *E. coli* isolates carried ESBL-associated genes (*bla*_TEM_, *bla*_SHV_, and *bla*_CMY-2_. However, only four isolates (M-W22UD LF3, M-W22UD LF7, M-W24UD LF3, and M-W29UD LF1) presented the ESBL phenotype and all of these carried *bla*_TEM_ and *bla*_CMY-2_. These results imply that the presence of drug-resistant strains of non-pathogenic *E. coli* isolates from the environment is possible. This can pose a threat to mastitis management programs for farm since one study also reported that non-pathogenic *E. coli* can serve as a reservoir of antibiotic resistance genes and could possibly transfer the genes to other pathogenic *E. coli* if conditions are suitable (Hu et al., 2016).

## Conclusion

This study provides evidence that *E. coli* isolates from cows with subclinical mastitis and from water at dairy farms in Saraburi Province of Thailand consisted of pathogenic *E. coli* strains that are resistant to many groups of antibiotics, including the fluoroquinolone group, which should raise concerns regarding the improper use of antibiotics in this area. However, the information on which antibiotics are being used on the farms is very limited. Identification of the ESBL phenotype and *β*-lactamase genes was also a concern as these can be transferred to other *E. coli* strains, including pathogenic strains, and bacterial species. This could lead to more serious problems associated with antibiotic resistance in the future. It should be recommended that farms prevent mastitis by promote clean environments for cows such as frequently changing bedding at the stalls and milking areas by cleaning the areas thoroughly. The use of dry and clean cloths to clean the teats before milking and effective teat dips should reduce mastitis on farms. The use of antibiotics, mastitis control programs, and milking hygiene should be considered and supervised by veterinarians to improve mastitis status and treatment in this area.

##  Supplemental Information

10.7717/peerj.3431/supp-1 Supplemental Information 1 Raw data for drug susceptibility testing

## Additional Information and Declarations

### Data Availability

The following information was supplied regarding data availability: The raw data has been supplied as a Supplementary File.
